# Analysis of leaf morphology, secondary metabolites and proteins related to the resistance to *Tetranychus cinnabarinus* in cassava (*Manihot esculenta* Crantz)

**DOI:** 10.1038/s41598-020-70509-w

**Published:** 2020-08-26

**Authors:** Yanni Yang, Xinglu Luo, Wanling Wei, Zhupeng Fan, Tangwei Huang, Xiaolu Pan

**Affiliations:** 1 College of Agronomy, Guangxi University, Nanning, 530004 China; 2 State Key Laboratory for Conservation and Utilization of Subtropical Agro-Bioresources, Nanning, 530004 China; 3 Guangxi Institute of Botany, Guangxi Zhuangzu Autonomous Region and Chinese Academy of Sciences, Guilin, 541006 Guangxi China; 4 Nanning Irrigation Experiment Station, Nanning, 530001 Guangxi China

**Keywords:** Plant sciences, Plant stress responses, Biotic

## Abstract

Constitutive resistance of plant can be divided into physical and chemical barriers. Cassava (*Manihot esculenta* Crantz) is susceptible to mites, especially *Tetranychus cinnabarinus*. Although significant differences in the resistance to *T. cinnabarinus* are observed in different cassava cultivars, limited research has been done on the mechanism accounting for the resistance. The aim of this study was to explore the mechanism of resistance to *T. cinnabarinus* by comparing morphology, secondary metabolites and proteins in different cassava cultivars. The anatomical structure of leaves showed that the cassava cultivar Xinxuan 048 (XX048), which showed a stronger resistance to *T. cinnabarinus* in both greenhouse testing and three years field evaluation tests (2016–2018), had thicker palisade tissue, spongy tissue, lower epidermis and leaf midrib tissue compared to cultivar Guire 4 (GR4). Greenhouse evaluation demonstrated that originally these cultivars were different, leading to differences in constitutive levels of metabolites. The proteomic analysis of protected leaves in XX048 and GR4 revealed that up-regulated differentially expressed proteins (DEPs) were highly enriched in secondary metabolic pathways, especially in the biosynthesis of flavonoids. This study not only provides a comprehensive data set for overall proteomic changes of leaves in resistant and susceptible cassava, but also sheds light on the morphological characteristics of cassava-mite interaction, secondary metabolite defense responses, and molecular breeding of mite-resistant cassava for effective pest control.

## Introduction

Cassava (*Manihot esculenta* Crantz) is the most important energy-producing root crop in the tropics. It belongs to the Euphorbiaceae family and has high carbohydrate production potential and adaptability to diverse environments. Food energy supplied by cassava is ranked fourth, after rice, maize and sugarcane, with more than 800 million people who survive on cassava in the world^1–3^.

*Tetranychus cinnabarinus* has a short life cycle, rapid development, high fecundity, wide host range and became an important pest. It mainly resides in the underside of cassava leaves and sucks nutrient ts from the leaves, causing them to turn yellow prematurely. When damaged seriously (more than 76% of the leaf was damaged by mites), the affected leaves dry and fall off, which negatively impacts the growth or even the survival of the plant. *T. cinnabarinus* can reduce cassava production by 50 to 70%, sometimes even 100%^4^. At present, the use of pesticides is a common method to control *T. cinnabarinus.* This arthropod reproduces about 15 generations in a year, with generations overlapping with each other. Therefore, the use of pesticides is very inefficient and looking for an effective control of *T. cinnabarinus* has become one of the most important problems in modern cassava production^5–7^.

The interaction between plants and pests is one of the important factors affecting plant productivity in both artificial and natural systems^8^. The leaf tissues are often the main target for different pests. Plants have developed sophisticated basic defense systems. Constitutive defenses, which including physical and chemical barriers, are critical to plant survival. The physical barriers hinder or prevent pests from feeding on their hosts. Chemical defenses, mainly toxins and secondary metabolites, reduce palatability and affect pest’s growth, development and digestion. Secondary metabolites (terpenoids, cardenolides, alkaloids, furanocoumarins and phenolics) are organic compounds in plants, which are not necessary for plant growth, but essential for reducing plant palatability and a deterrent for pests^9^. According to Wu and Baldwin, plants produce approximately 500,000 secondary metabolites, most of which are powerful pest-resistant chemicals^10^. The level of secondary metabolites varies depending on plant species and plant tissues. It has been found that plant defense-related proteins increase after herbivore feeding and were correlated with secondary metabolites synthesis and signaling pathways^11,12^. Other studies also showed that the content of secondary metabolites increased remarkably in plant leaves infested by mites^13–16^.

The three major secondary metabolites of plants are phenolics, terpenoids, and nitrogenous organic compounds^17^. In plants, it is well established that phenolics can act as antioxidants by providing electrons to callus peroxidases, detoxifying the H_2_O_2_ produced under different stress conditions, including biological ones^18,19^. Moreover, phenolics can be toxic to insects and affect the feeding, growth, development and reproduction of *T. cinnabarinus*. Buffon et al.^20^ showed that the content of total phenolic compounds in the infested leaves of the tolerant cultivar rice varieties was higher than that of susceptible rice varieties.

Flavonoids such as flavones and tannin are important secondary metabolites in plants. They commonly accumulate in epidermal cells of plant organs such as leaves, flowers, stems, roots, seeds and fruits. Flavonoids have strong acaricidal activity against *T. cinnabarinus*, and further studies have confirmed that the accumulation of flavonoids was the key mechanism of sour orange plants against mites^21^. At present, little flavones metabolic research has been done in cassava, and perhaps such a key mechanism remains to be demonstrated. Tannin united with starch, can negatively affect the feeding and digestion of starch and other nutrients by insects^22^. Physiological indicators of secondary metabolites measured in cassava mainly include phenolics, flavones and tannins.

Proteomics have been widely applied in stress tolerance studies in plants^23–26^. Many studies with proteomic and gene expression analysis have shown that the responses to *T. cinnabarinus* are extremely complicated across different plant species and stages of growth^27–31^. In the case of resistance to *T. cinnabarinus* in cassava, another critical factor is the unique morphological structure of cassava^32^. The unique genotypes include luxurious and long hairs on their underside leaf veins, thick hypodermis and spongy tissues and low cytochylema osmotic pressure. These had comparatively high acarid resistance, for the mites fed with them grew slowly and laid fewer eggs^33^. To date, few studies have been reported on cassava responses to *T. cinnabarinus* through combining morphological structure, physiology and proteomics.

In this study cassava genotypes XX048 and GR4 (mite-resistant and susceptible, respectively), were evaluated through long-term (2016–2018) field experiments. The anatomy, physiology and proteomics of these two cassava cultivars were analyzed in response to *T. cinnabarinus* feeding. These results will expand our understanding in the mechanism of cassava resistance to *T. cinnabarinus*, which will further provide new insights for cassava breeding.

## Results

### Field evaluation of the damage caused by *T. cinnabarinus* in two cassava genotypes

Two cassava clones were planted continuously from 2016 to 2018 in areas seriously affected by *T. cinnabarinus* without the application of any pesticide. Field examinations were conducted to evaluate the reaction to *T. cinnabarinus* of the two genotypes. As shown in Table 1, there were significant differences in the mite damage index and population densities of *T. cinnabarinus* of the two cassava clones. The mite damage index of XX048 from 2016 to 2018 was 49.67%, 42.42% and 49.69%, respectively, while that of GR4 was 70.09%, 86.40% and 90.90%, respectively. The correlation coefficient of the population density and the damage index was 0.985 (P < 0.01). The mite damage index of XX048 remained stable in 3 years, while that of GR4 increased year by year. We speculate that the decrease of secondary metabolites may lead to the increase of mite damage index. The highest population density of XX048 over three years was in 2018, which may be related to the decrease of total phenols content.

**Table 1:** Field evaluation of the damage caused by *T. cinnabarinus* in two cassava genotypes from 2016 to 2018.

Parameter	2016		2017		2018	
	XX048	GR4	XX048	GR4	XX048	GR4
Mites damage index (%)	49.67 ± 8.58	70.09 ± 2.25	42.42 ± 7.69	86.40 ± 5.30	49.69 ± 7.96	90.90 ± 5.75
Resistance grade	MR	S	MR	S	MR	HS
Population density (mites/leaf)	10.9 ± 2.47	38.33 ± 5.38	10.65 ± 4.23	46.1 ± 3.89	15.33 ± 3.63	56.2 ± 6.28

These numbers in the table are presented the mean ± standard deviation (SD) based on three biologically independent values. MR, S, and HS stand for “mite-resistant”, “mite-susceptible”, and “highly mite-susceptible”, respectively.

Based on the field evaluation, the *T. cinnabarinus* resistance grade for XX048 and GR4 cassava cultivars were categorized as mite-resistant and mite-susceptible (highly mite-susceptible), respectively.

### Comparison of leaf anatomical structures in different cassava genotypes

**Figure 1:**
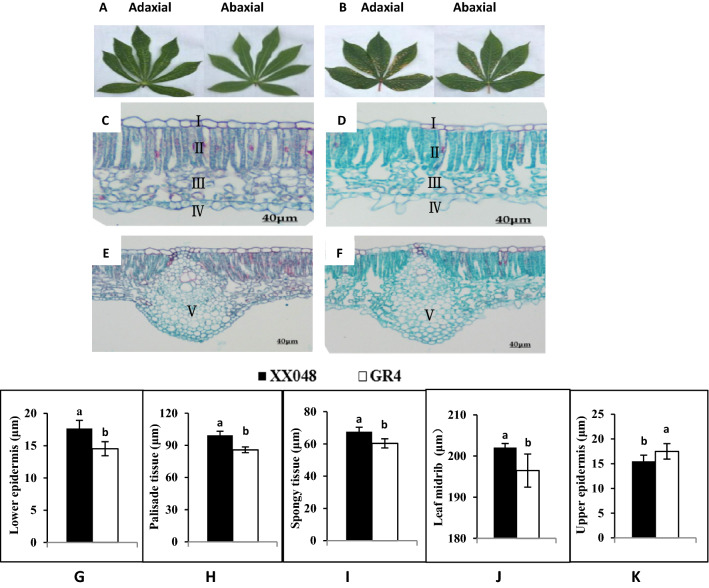
Comparison of leaf morphological and anatomical characteristics of the leaves from different cassava cultivars. (**A**,**B**) pictures of both sides of the cassava leaf from XX048 (**A**) and GR4 (**B**); (**C**,**D**) transverse sections of XX048 (**C**) and GR4 (**D**) leaves; E and F, leaf midrib of XX048 (**E**) and GR4 (**F**) leaves. (**C**,**D**: 10 × 40; **E**,**F**: 10 × 20). I: Upper epidermis II: Palisade tissue III: Spongy tissue IV: Lower epidermis V: Leaf midrib. These values are presented as the mean ± standard deviation (SD) based on three biologically independent values. Letters above the histogram indicate the statistical significance (p < 0.05).

Differences in the anatomical structures of cassava leaves were also investigated. Figure 1 shows the leaves’ morphology and the transverse sections of the leaves XX048 and GR4. Compared with GR4, the thickness of lower epidermis, palisade tissue, spongy tissue and leaf middle of XX048 increased by 21.52%, 15.77%, 10.15% and 11.96%, respectively, while the upper epidermis was decreased by 11.49% (Fig. 1G–K). The above values of the two genotypes were significantly different (P < 0.05). The most noticeable difference between the genotypes was in the thickness of the lower epidermis and palisade tissue (Fig. 1G,H). This may be the unique morphological structure of XX048 genotype, which makes it more resistance to *T. cinnabarinus*.

### Comparison of secondary metabolites content in leaves of XX048 and GR4 affected by *T. cinnabarinus* in the field

**Figure 2:**
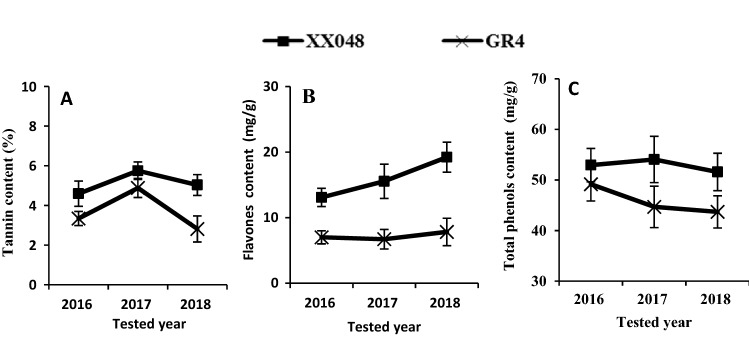
Comparison of secondary metabolites content in leaves of XX048 and GR4 affected by *T. cinnabarinus* from 2016 to 2018 in the field.

The tannin, flavones and total phenols contents were measured and compared in the two mite-infested clones by *T. cinnabarinus* from 2016 to 2018. As shown in Fig. 2, XX048 had synthesized more secondary metabolites in the leaves than GR4. Tannin content increased from 2016 to 2017 and gradually declined from 2017 to 2018 (Fig. 2A). Flavones increased gradually from 2016 to 2018 in XX048, while remaining at similar levels in GR4 throughout the time (Fig. 2B). The level of total phenols remained stable in the leaves of XX048, while significantly decreased in GR4 over the three years (Fig. 2C). As the experiment didn’t have a control, the two cultivates might be equally susceptible to mites. But in fact, XX048 was more resistant to mites than GR4. At the same time, according to the results of field experiments for 3 years, XX048 clearly accumulated higher levels of secondary metabolites, which potentially contributed to its higher resistance to the mite. However, this needed to be confirmed by molecular technology.

### Comparison of secondary metabolites content in leaves protected or affected by *T. cinnabarinus* of in greenhouse

**Figure 3:**
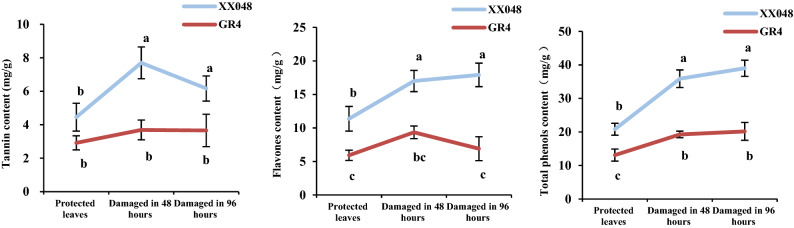
Comparison of secondary metabolites content in leaves protected or affected by *T. cinnabarinus* in greenhouse. These values are presented as the mean ± standard deviation (SD) based on three biologically independent values. Letters above the histogram indicate the statistical significance (p < 0.05).

Tannin, flavones, and total phenol contents in leaves from XX048 and GR4 was also measured in plants grown in the greenhouse. Metabolites were measured in plants protected or infested (12 mites per leaf) with *T. cinnabarinus*. As shown in Fig. 3, compared with the protected leaves of GR4, the content of tannin, flavones and total phenols in XX048 clones increased by 52.55%, 92.06% and 58.78%, respectively. The results showed that the secondary metabolites of XX048 increased rapidly in the presence of mites. However, GR4 did not show the same difference in tannin and flavones contents. The results above indicated that the synthesis of secondary metabolites in plants from the two clones grown in the greenhouse was significantly different even in protected leaves.

Field and greenhouse evaluation confirmed not only that XX048 was more resistant to *T. cinnabarinus* than GR4, but also that there were differential constitutive levels of metabolites. Moreover, differences in metabolites were enhanced 48 h after challenging plants with the mite.

### Identification and quantification of differentially expressed proteins

**Figure 4:**
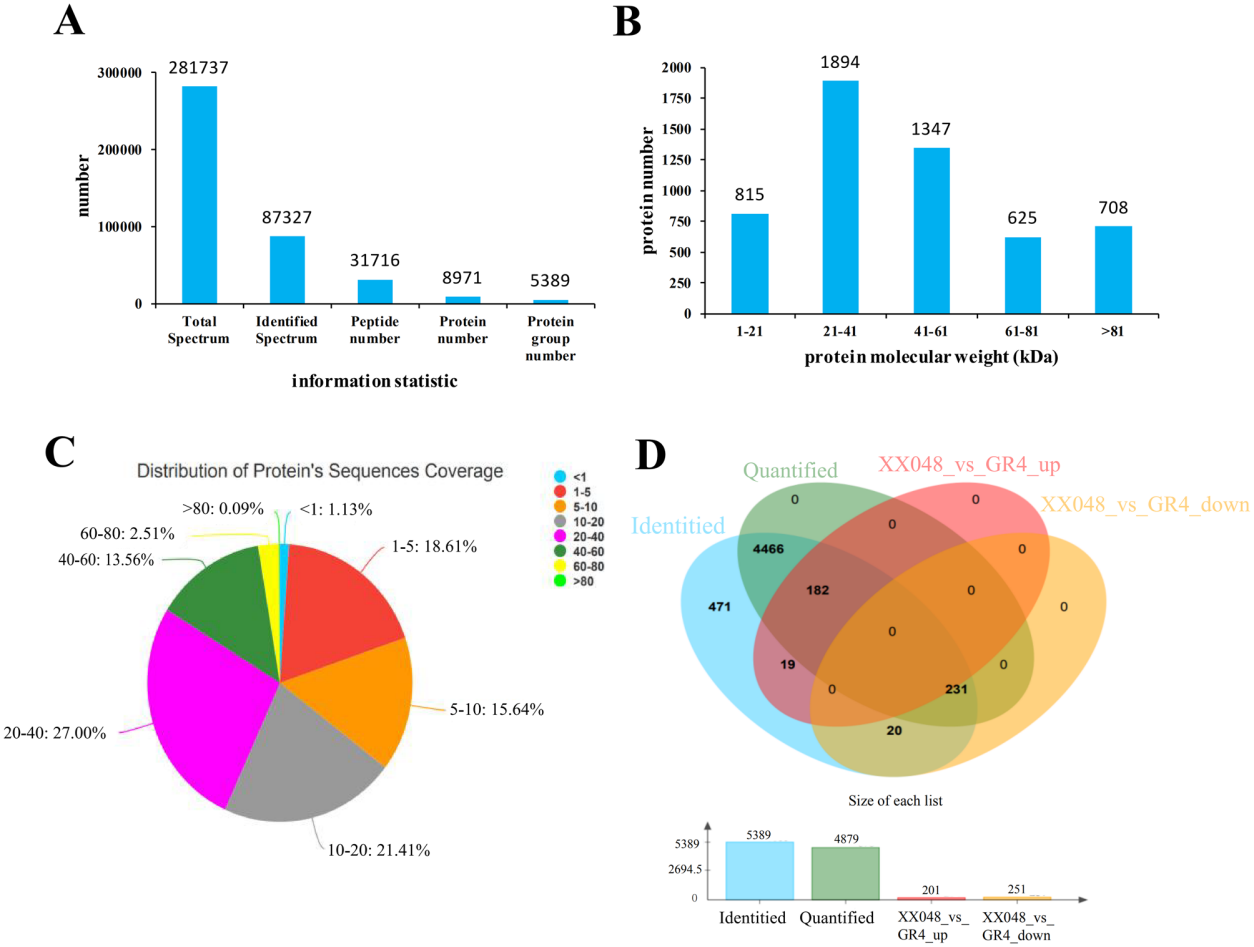
General information of the identified proteins. (**A**) The protein information; (**B**) The distribution of protein molecular weight; (**C**) The peptide length distribution coverage; (**D**) Venn diagram showing quantitative distribution of differentially expressed proteins in the samples of XX048 and GR4, respectively.

Differentially expressed proteins (DEPs) were explored in protected leaves of the two cassava genotypes. Using iTRAQ labeling LC–MS/MS analysis, a total of 281,737 spectra were acquired and 87,327 of them matched to the reference spectra. There were 31,716 peptides and 8,971 proteins in three independent experiments with a 1% false discovery rate (FDR). Resulting mass spectrometry data have been deposited into the iProX (https://www.iprox.org) with the identifier IPX0001727000. The general information is presented in Fig. 4A. A total of 5,389 proteins were identified. Among these proteins, 815 were between 1 to 21 kDa, 1,894 between 21 to 41 kDa, 1,347 between 41 to 61 kDa, 625 between 61 to 81 kDa and 708 over 81 kDa (Fig. 4B). The peptide length distribution coverage of most of the identified proteins ranged from 5 to 20 (Fig. 4C). Student’s t-test was applied to determine if the proteins in the XX048 and GR4 samples were significantly different based on 4,879 quantified proteins (p < 0.05). According to the fold change (FC) ≥ 1.20 or ≤ 0.83 and p < 0.05, 452 differentially expressed proteins (DEPs) demonstrated dynamic changes between the two cultivars, with 201 DEPs up-regulated and 251 DEPs down-regulated (Fig. 4D).

Using the pHEATMAP package in R, hierarchical clustering was performed to show a comprehensive overview of the DEPs (Figure S1). These results showed that the three replicates of each sample from each clone were clustered into the same respective clades, demonstrating the reproducibility and reliability of our LC–MS/MS data. It is worth noting that the clustering results based on the mean of protein abundance showed significant differences between the two cassava genotypes, indicating that they mobilized a large amount of proteins and differentially regulated their abundance to different degrees.

### GO annotations and functional classifications analysis of DEPs

To further reveal the functions of the identified DEPs, GO functional classification analysis was performed. In the GO analysis, the DEPs were analyzed using three sets of ontologies: the biological process, the cellular component and molecular function (Figure S2). The biological process analysis revealed three top prominent processes: metabolic, single-organism and cellular. These results indicate that the metabolic processes were significantly different among varieties, which may determine the resistance to *T. cinnabarinus*. The cell, cell parts and organelle categories were three cellular compartments in which DEPs were highly localized. In terms of molecular function, the largest group was protein with catalytic activity and the second largest group identified involved binding proteins.

GO enrichment based on DEPs analyses was performed by dividing all quantified proteins into four quantiles according to the fold change (FC) ratio in this study: FC4 (> 1.5), FC3 (1.2–1.5), FC2 (0.67–0.83) and FC1 (< 0.67) (Table S1–S4). This analysis was carried out to better understand the preferred functional characteristics for the DEPs. In the biological process category, proteins involved in the anthocyanin-containing compound biosynthetic process, flavonoid biosynthetic process and secondary metabolite biosynthetic process were found to be significantly enriched in FC4 (> 1.5). On the other hand, proteins involved in carbohydrate metabolism, oxidation–reduction and protein phosphorylation processes were highly enriched in FC1. In the cellular component category, the up-regulated DEPs proteins were significantly enriched in the integral component of the membrane. Molecular function showed that the proteins with hydrolase activity were enriched in FC4, and the proteins with transferase activity were enriched in FC1.

These results, therefore, indicated that DEPs of XX048 and GR4 were involved in diverse biological processes. The proteins upregulated in clone XX048 were more concentrated in the flavonoid and secondary biosynthetic processes.

### Protein domain analysis of the DEPs

**Figure 5:**
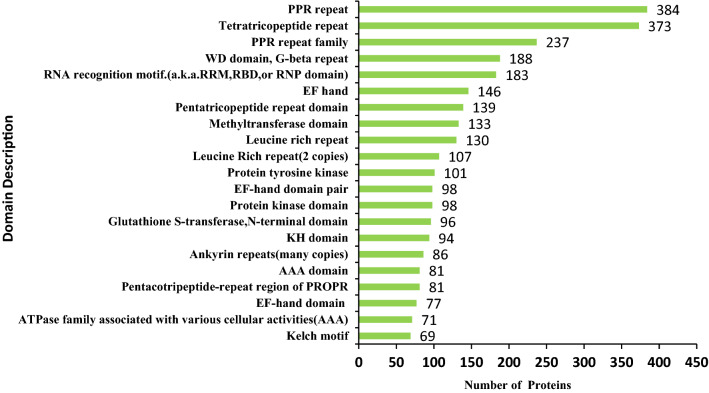
Protein domain analysis of the DEPs.

To investigate the DEPs domain features of XX048 and GR4 genotypes, domain annotation analysis was performed. Results showed that pentatricopeptide repeat (PPR) domain and tetratricopeptide repeat (TPR) domain were highly enriched (Fig. 5). Further cluster analysis indicated that glutathione S-transferase (GST), short chain dehydrogenase (SDR) and ATPase family-associated proteins with various cellular activities were highly enriched in up-regulated proteins (Table S5). Among the down-regulated proteins, RNA recognition motif and N-terminus of xylanase inhibitors were found to be highly enriched (Table S6). The results show that the two clones altered protein expression with various structural domain characteristics in response to DEPs.

### KEGG enrichment and pathway analysis of the DEPs

**Figure 6:**
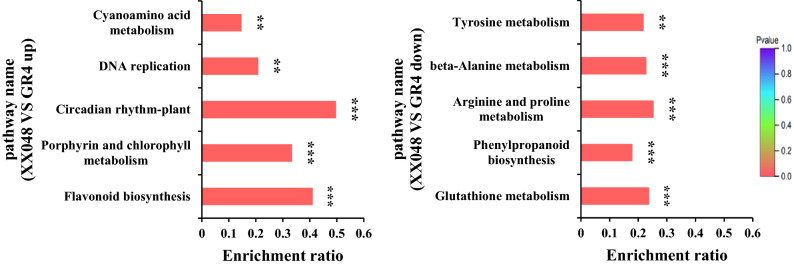
KEGG enrichment analysis of the DEPs in XX048 and GR4 cassava leaves.

Kyoto Encyclopedia of Genes and Genomes (KEGG) pathway analysis of the DEPs was performed based on enrichment to gain deeper insight into the pathways regulated by the two genotypes (Fig. 6). The up-regulated proteins were significantly enriched in flavonoid biosynthesis (Figure S3), porphyrin and chlorophyll metabolism, circadian rhythm, DNA replication and cyanoamino acid metabolism. The down-regulated proteins were significantly enriched in glutathione metabolism, phenylpropanoid biosynthesis, arginine and proline metabolism. These results indicate that the DEPs from XX048 and GR4 were highly associated with the flavonoid biosynthesis.

### Protein–protein interaction (PPI) network analysis of the DEPs

DEPs (FC4 > 1.2 and FC1 < 0.83) against the String database were searched for, and a PPI network was constructed to further understand the association between cassava cultivars resistance and protein–protein interactions. Since there is no PPI data in the String-database of cassava proteins, we used homologous proteins with *Arabidopsis thaliana* as reference (Table S7).

**Figure 7:**
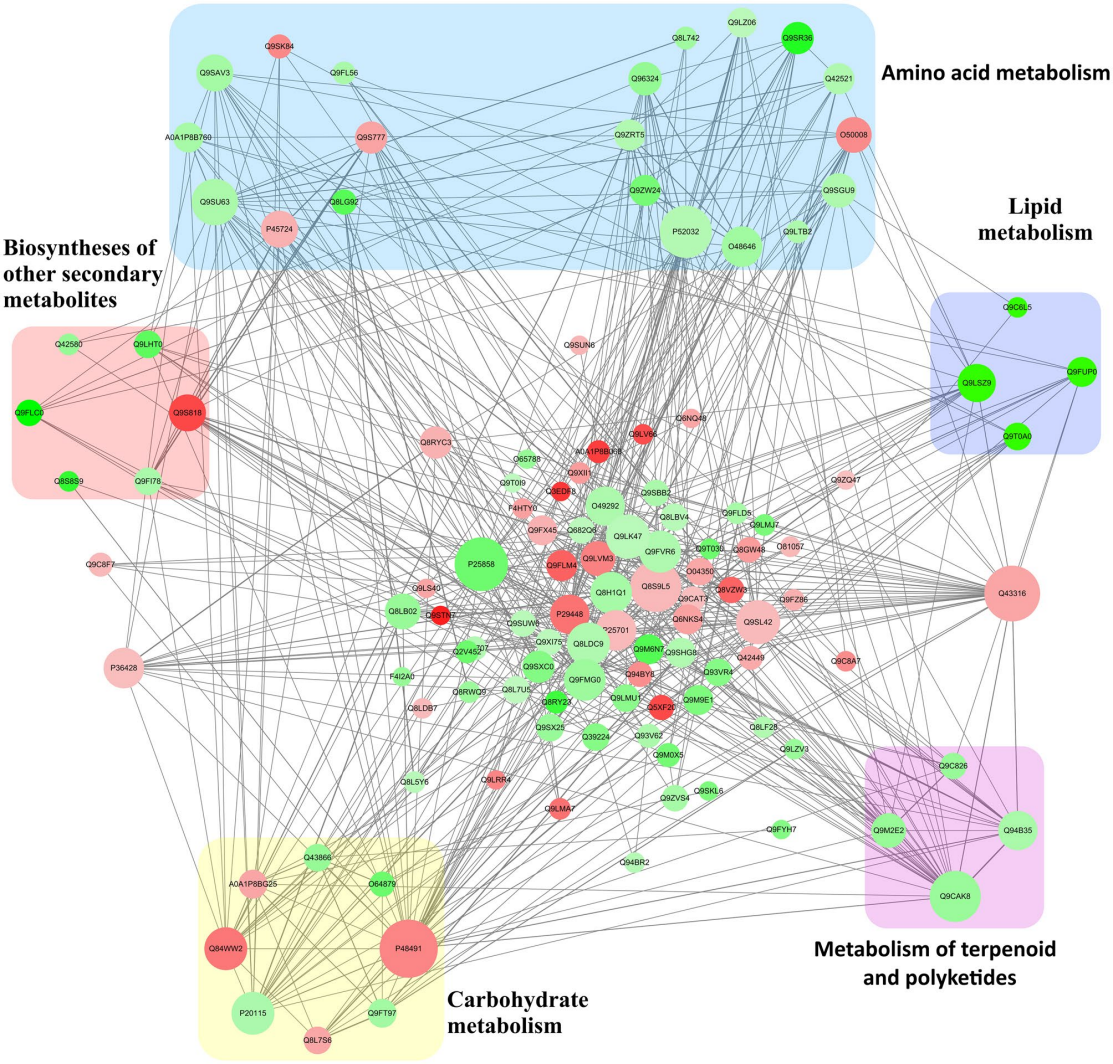
PPI network analysis of DEPs in XX048 and GR4 cassava leaves. DEPs related to amino acid metabolism, biosynthesis of other secondary metabolites, carbohydrate metabolism, metabolism of terpenoids and polyketides and lipid metabolism were indicated in backgrounds of blue, red, yellow pink and purple, respectively. Each node in the figure represents a protein, and the size of nodes indicates the degree of correlation between proteins. Each line indicates the interaction between proteins. The green and red dots represent down-regulation and up-regulation, respectively.

The global network diagram of these DEPs interactions is presented in Fig. 7. Many proteins were involved in multiple interactions. The top protein–protein interaction networks that stood out from the analysis as significantly enriched were the ones related to amino acid metabolism, biosynthesis of other secondary metabolites, carbohydrate metabolism, metabolism of terpenoids and polyketides and lipid metabolism.

### Validation of gene expression of the DEPs

**Figure 8:**
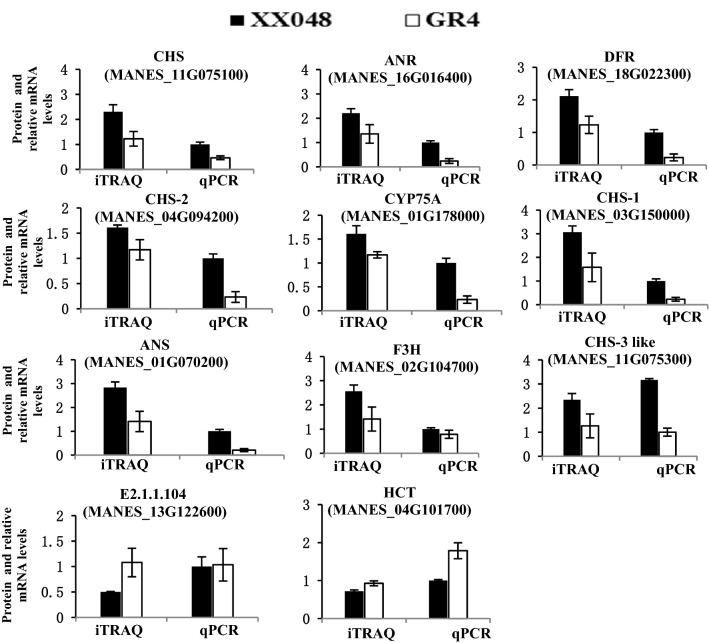
Relative expressions of 11 genes at the protein and mRNA levels.

Eleven DEPs associated with flavonoid biosynthesis were selected for qPCR analysis to confirm the correlation of proteins and their corresponding mRNAs expression profiles (Table S8). The qPCR results showed the expression profiles of the 11 genes (Manes.11G075100, Manes.16G016400, Manes.18G022300, Manes. 04G09420, Manes.01G178000, Manes.03G150000, Manes.01G070200, Manes.02G104700, Manes.11G075300, Manes.13G122600, Manes.04G101700) were in agreement with the iTRAQ data (Fig. 8). The consistent expression profiles of qPCR and iTRAQ indicates that the expression of these genes changed from the transcription level.

## Discussion

The two cassava genotypes had differential reactions to *T. cinnabarinus*. Understanding the mite effect on different clones and the main regulatory genes is important for breeding *T. cinnabarinus*-resistant cultivars. Both mites damage index and population density can serve as important indicators of the resistance in XX048 to this mite. Previous studies reported mite resistance strategies that involve external morphological and internal tissue structure as well as the action of metabolites on mites^34^. Therefore, plant resistance can be divided into two categories: physical and chemical barrier. Physical barriers include cell wall modifications, thorns, trichomes, spines and surface waxes to hinder or deter herbivores accessibility. The production of a chemical barrier, such as latex, oils, resins, sticky compounds and secondary metabolites reduce palatability and affect growth, development and digestion of herbivores.

The physical barrier on the leaves is the first line of defense against the mites. In this study, light micrograph of leaves showed that thicker lower epidermis, palisade tissue, spongy tissue, leaf midrib thickness and thinner upper epidermis in XX048, compared to GR4, may be contributing factors to XX048 having higher resistance to *T. cinnabarinus* than GR4. Particularly, there were significant differences in the thickness of the lower epidermis and palisade tissue (Fig. 1). The mite damage index of cassava cultivars was negatively correlated with lower epidermis and palisade tissue, indicating that the varieties with thicker lower epidermis and palisade tissue would be expected to have a low mite damage index and strong resistance. Sun et al.^35,36^ also reported that one of the structural characteristics of stress-resistant plant leaves was the well-developed palisade tissue, which is consistent with the results of this experiment. Moreover, since *T. cinnabarinus* often infects the abaxial surface of leaf, the tissues cells of the lower epidermis can be used as an anatomical and structural indicator for the *T. cinnabarinus* resistance of cassava.

Among the chemical defenses, secondary metabolites, especially tannin, total phenols and flavonoids were linked to the reaction to the mite. War et al. reported that the secondary metabolites in leaves were involved in plant defense responses to *T. cinnabarinus*^37^. Plant-generated secondary metabolites can be strong repellents to pests, and therefore help defend the plants against *T. cinnabarinus*. In this study, field experiments conducted from 2016 to 2018 showed the most substantial increase in flavones in XX048 among the secondary metabolites, while the content of GR4 in the same period did not increase significantly (Fig. 2). This strong connection suggests that XX048 mite-resistance could be due to the synthesis of flavonoids.

In the greenhouse experiment, the secondary metabolite content in the two cassava clones were significantly different even in protected leaves. It was possible that originally these cultivars were different, leading to different constitutive levels of metabolites. And the contents of secondary metabolites in different clones may be closely related to plant growth and resistance, which needs to be molecularly confirmed.

Flavonoids constitute a relatively abundant family of aromatic molecules synthesized from phenylalanine (Phe) and malonyl-coenzyme A (CoA; through the fatty acid pathway)^38^. These compounds comprise six major subgroups found in most higher plants: chalcones, anthocyanins, flavones, flavonols, flavandiols and condensed tannins (or proanthocyanidins); the seventh group are aurones which are widespread but not ubiquitous. In recent years, many efforts have been made to understand the flavonoids biosynthesis pathway from the level of molecular genetics. Flavonoids play important roles in signals between plants and microorganisms and in male fertility of certain species. They are also antimicrobial agents and feed inhibitors and serve a key role in UV protection. The "early" steps in this pathway have even been found in bryophytes (mosses). It has been suggested that the synthesis of flavonoids compounds may have evolved first to provide chemical messengers, and then to provide UV sun protection^39^. In this paper, a majority of proteins related to secondary metabolism increased with FC > 1.5 in XX048 (Table S4), but not in GR4, indicating that XX048 had different secondary metabolite synthesis than GR4. Therefore, revealing the differentially expressed proteins and the main regulatory genes in the metabolism of the secondary products could provide insights for breeding resistance clones to *T. cinnabarinus*.

Glutathione S-transferase (GST) represents a superenzyme encoded by several genes and possesses multifunctions^40,41^. It is also a major group of detoxification enzymes. Evidence suggests that the level of GST expression is a key factor in determining the sensitivity of cells to many toxic chemicals^42^. Yusuf confirmed that glutathione S -tranferase (GST) and its activity were important oxidative biomarkers affected by the antioxidants supplementation (p < 0.05)^43^. The increase of GST level was correlated to the resistance to oxidative damage. GST plays an important role in the identification and transportation of anthocyanins into plant cell vacuoles^44,45^. During development and in response to stress, reactive oxygen species (ROS) are produced from lipid peroxidation caused by the death of plant cells^46^. Therefore, increasing the expression of antioxidative enzymes to regulate the cellular redox state may be one of the detoxification mechanisms against ROS. GST is an important component of the ascorbate–glutathione cycle. The first phase of the ascorbate–glutathione cycle is driven by GST, which plays an important role in the regeneration of ascorbate and detoxification of lipid peroxidation byproducts by binding glutathione to the lipid alkoxyl radical^47^. In our study, the GST protein in cassava leaves was highly expressed in XX048. Valtaud et al., confirmed that the expression of GST was an early indicator of plant disease and could be used as a stress marker^48^. This finding is consistent with the proteomic analysis on other plants responding to mite attack.

Short chain dehydrogenase (SDR) is an ancient superfamily of enzymes and a large family of proteins that can participate in the metabolism of different specific substrates by oxidoreduction, isomerization and cleavage. Many studies showed that SDR is mainly involved in the growth and various endogenous and exogenous metabolism of substances (poisons) of the organism. Previous studies found that the gene expression level of SDR is significantly higher in a fenpropathrin resistant strain to *T. cinnabarinus*, suggesting that the resistant strains of *T. cinnabarinus* may be related to SDR^49^. In 2010, when studying the pathogenic mechanism of Magnaporthe oryzae, it was found that SDR could not only regulate the pathogenicity of Magnaporthe oryzae, but also regulate the overall defense ability of plants^50,51^. Tonfack et al. founded the SDR superfamily involvement in primary and secondary metabolism in plant^52^. In the current study, expressions of SDR were increased in XX048, while GR4 were decreased. These findings suggest that SDR may play an important role in the resistance of different cassava varieties to *T. cinnabarinus.*

ATPase family associated with various cellular activities (AAA) proteins commonly exist in prokaryotes and eukaryotes, and are involved in diverse cellar functions including protein degradation, membrane fusion and vesicle transport, cell organelles formation and maintenance^53^. They are also critical for the mitochondrial function, cell cycle, proteolysis and peroxisome assembly^54^. In this study, the abundance of AAA proteins increased in the cassava of XX048 in response to *T. cinnabarinus*. Different components of the AAA protein showed different regulation mechanisms, which regulate the protein synthesis associated with the tolerance of *T. cinnabarinus* in different cultivars.

KEEG enrichment pathway analysis showed that flavonoid pathway was highly enriched in XX048. The findings reinforce that flavonoid biosynthesis pathway may play a vital role in resisting against *T. cinnabarinus*. Yang et al. speculated that the resistance of mite might be related to the biosynthesis of three secondary metabolites: flavonoids, glutathione and phenylpropanoid^55^. This study shows the consistent association between the resistance of cassava to mites and the flavonoid biosynthesis pathway, which awaits functional study.

The expressions of the 11 flavonoid biosynthesis genes were completely consistent with their corresponding proteins, indicating that there was a strong correlation between gene transcription levels and their protein abundance in this study. These findings suggest that many DEPs in the flavonoid biosynthesis pathway were involved in regulation under the *T. cinnabarinus* condition. These results have expanded the understanding of the molecular mechanism of cassava resistance to the mite. Further study on the function of each identified protein will be valuable.

## Conclusion

Plant resistance can be divided into two categories: physical barriers and chemical barriers. Using morphological, physiological and iTRAQ-based proteomics analysis methods, we compared the mite-resistant variety XX048 and mite-susceptible variety GR4*.* Morphological structure indicated that lower epidermis and palisade tissue may be the key physical barriers leading to XX048 being more resistant to *T. cinnabarinus* than GR4. Physiological results showed that the flavonoids content in different clones are closely related to plant growth and resistance. In our iTRAQ-based proteomics results, 452 DEPs were identified in XX048 and GR4. Strikingly, anthocyanin-containing compounds, flavonoid and secondary metabolite biosynthetic processes were found to be significantly enriched in XX048 up-regulated proteins. These results indicate that the flavonoid biosynthesis pathway is one of the most effective and highly selective protein secondary metabolites pathways in cassava variety XX048. The qPCR results showed that the expression profiles of 11 flavonoid biosynthesis genes were in agreement with the iTRAQ data, which indicated that the transcription level of genes were parallel to the corresponding protein level. These data help understand the complexity of resistant to *T. cinnabarinus* proteins, and the screening of DEPs will contribute to further functional studies. These results will greatly improve the knowledge of the molecular mechanism of *T. cinnabarinus* in cassava.

## Methods

### Materials

Cassava clone ‘Xinxuan 048’ (XX048) is a new cultivar which was selected by the cassava research group of the Agricultural College, Guangxi University (GXU), by the systematic breeding method from cassava resource ZM93-16 with natural variation. ‘Guire 4’ (GR4) is a bred genotype obtained from an open-pollination (half-sib family SM1600) from MPAR164 (introduced from the International Center for Tropical Agriculture). The collection of cassava germplasm was done by Dr. Xinglu Luo and deposited in the cassava resources conservation field of the Agricultural College, Guangxi University, complying with legislation of China.

### Cultivation of cassava

#### Field cultivation

The field cultivation took place at the experimental station of Agriculture College, Guangxi University (ESAC) from 2016 to 2018.

The row spacing was 0.9 m × 1.0 m, and the plot area was 16.2 m^2^. Completely random block design, with three repetitions, was adopted. Before planting, 15,000 kg of organic fertilizer and 225 kg of compound fertilizer (N-P_2_O_5_-K_2_O) per hectare were applied as base fertilizer. Sixty days after planting, a topdressing (150 kg of urea, 225 kg of compound fertilizer and 112.5 kg of KCl per hectare) was applied. Routine cultural management was used. No agrochemicals were used throughout the planting process, allowing cassava to be naturally infested with *T. cinnabarinus*.

#### Greenhouse cultivation

Stems cuttings were planted inside a gauze element cages within a greenhouse. In total, about 800 kg topsoil (0–20 cm) was collected from ESAC. Stem cuttings from XX048 and GR4 were planted in pots (39 cm × 58 cm × 40 cm), containing 5 kg of homogenized soil (organic manure is mixed with the topsoil in a ratio of 1:5) and grown in the greenhouse (13 h/11 h of light/dark, 28 °C ± 2 °C of day/night). Each cassava cultivar was divided into protected cassava and infested cassava. The growth was observed every day to ensure that no mites or pests were present. Every treatment included three replicates with twelve plants each. Different replicates were planted in different areas of the greenhouse, separated by a mesh. Each plot planted with two stem cuttings were planted in each pot and one shoot per plant was kept for the experiment.

### *T. cinnabarinus* rearing

Healthy *T. cinnabarinus* adults were collected from cassava fields at ESAC. After identification, the mites were reared on the underside of fresh cassava leaves with careful observation to make sure there were no other impurities. Leaves and mites were placed on a pasteurized, water-soaked sponge and placed in glassware about 30 cm in diameter and 10 cm in height. The leaf margin was wrapped with water-saturated paper to prevent mites from escaping. Mite-infested leaves were kept under the following conditions: temperature 28 ± 1 °C, 70 ± 5% relative humidity (RH), and 14 L:10 D photoperiod prior to the experiments. Fresh leaves were replaced every 2 days.

### Field evaluation

Six fully-expanded leaves per plant (two leaves from each top, middle and bottom sections of the plant) were chosen. Field resistance evaluation was performed based on a published protocol^56^. According to symptoms from the damage by *T. cinnabarinus* on the leaves, the scale of damage was divided into five scales as follows:

0: The leaves were not damaged by mites and the plants grew normally.

1: Less than 25% of the leaf was damaged by mites.

2: 26–50% of the leaf was damaged by mites.

3: 51–75% of the leaf was damaged by mites.

4: More than 76% of the leaf was damaged by mites.

The field response of cassava host to *T. cinnabarinus* was divided into six levels (Table S9). The mites damage index activity and population density were calculated by the following formula:

mites damage index (%) = [∑ (the damage scale of cassava leaf × the number of leaves of the damage scale) / the total number of leaves investigated × the highest damage scale] × 100.

Population density (mites / leaf) = total number of live mites / total number of leaves screened.

### Sampling and processing of leaves

Cassava was grown in the field and leaf samples were collected 150 days after planting during the stage of root tuber expansion. In the greenhouse, cassava leaves were sampled three months after planting. The fourth and fifth leaf samples were frozen in liquid nitrogen immediately and stored at − 80 °C for further determination. Other fresh leaves (three leaves from each section, i.e., top, middle and bottom) were sampled and dried to measure the content of tannins, flavones and total phenols.

### Secondary metabolite physiological parameters

The content of secondary metabolites was measured in oven-dried leaf samples. Content of tannin, total phenol and flavonoids were analyzed according to Gao^57^, Yin et al.^58^ , Zhang and Cao et al.^59,60^.

### Morphological observation under light microscopy

Morphological analysis under light microscopy of XX048 and GR4 leaves was conducted as described in An et al*.*^61^. The thickness of lower epidermis, palisade tissue, spongy tissue, leaf midrib and upper epidermis were measured. The third completely expanded leaf of the upper part of the plant was selected and 0.5 cm^2^ leaf blocks were cut on both sides of the middle vein. Twenty such leaves were analyzed for each genotype, and each leaf was measured three times.

### Proteomic analysis

Two protected samples from 3-month-old XX048 and GR4 plants grown in the greenhouse were taken for proteomic analysis. Among the three replicates of each clone, a total of 12 fully expanded leaves were selected and homogenized into a bulk sample. The process of total protein extraction, trypsin digestion, peptide desalting, high-resolution LC–MS/MS analysis, iTRAQ labeling protein identification and quantification were conducted as described Cen et al*.*^62^.

### RT-PCR analysis

Leaf samples of XX048 and GR4 from 3-month-old plants grown in greenhouse were also taken for qRT-PCR analysis. Total RNA was isolated from the samples by using the HUAYUEYANG RNA extraction kit (a biotechnology company in Beijing, China), cDNA was performed by reverse transcription from 1 μg total RNA with the Takara cDNA Synthesis Kit (a biological engineering company in Dalian, China) and primers were designed for qPCR using primer 5.0 (Table S8). The cassava TAF 15b gene was used as an endogenous reference for qPCR^63^. The relative expression of the primers designed for qPCR detection was selected by DEPs at the transcript level. The PCR was mixed by Roche Lightcycler 480 Real-time PCR System in 10 μL with SYBR Green PCR reactions Master Mix kit (Vazyme), following the manufacturer’s protocol. These reactions were performed with three replications. The relative amount of each gene expression levels was calculated using the 2 ^−ΔΔCT^ method^64^.

### Bioinformatics and data analysis

All the mass spectrometry data have been deposited into the iProX (https://www.iprox.org) with the identifier IPX0001727000. These peptides were identified using Sequest software integration from Proteome Discoverer (version 2.1, Thermo Fisher Scientific). Trypsin was selected as enzyme to allow two missed cleavages. Carbamidomethylation (C) was set as a fixed modification. In addition, Oxidation (M) and acetylation in N-Term were set as variable modification. The searches were performed using a peptide mass tolerance of 20 ppm and a production tolerance of 0.05 Da, resulting in a 1% false discovery rate (FDR).

The R statistical software v. 2.10.0. was used to detect DEPs. DEPs of significance had an FC < 0.83 or > 1.2, and a Student’s t test P < 0.05. Blast2 GO (v. 2.5.0) was used for functional annotation of proteins, Goatools (v0.6.5) for GO enrichment analysis, and Python for KEGG enrichment analysis. A protein–protein interaction (PPI) network of DEPs was constructed in the String database. Since cassava data were not available, *Arabidopsis thaliana* was used as a reference. ANOVA was used for statistical analysis, and SPSS 18.0 (SPSS Science, Chicago, IL, USA) was used for statistical analysis to Duncan’s tests. A value of P < 0.05 was considered a statistically significant difference.

## Supplementary information

Supplementary figures.

Supplementary tables.
