# Supplementary material for: Analysis of leaf morphology, secondary metabolites and proteins related to the resistance to Tetranychus cinnabarinus in cassava (Manihot esculenta Crantz)
Source: Sci Rep. 2020 Aug 26;10:14197. doi: 10.1038/s41598-020-70509-w (PMC7450062; doi:10.1038/s41598-020-70509-w)
Supplement: Supplementary file 1 — Supplementary figures. [file 41598_2020_70509_MOESM1_ESM.docx]

**Analysis of leaf morphology, secondary metabolites and proteins related to the resistance to *Tetranychus cinnabarinus* in cassava (*Manihot esculenta* Crantz)**

**Figure supplementary information**

Yanni Yang^1^, Xinglu Luo^1,2^,Wanling Wei^1,3^, Zhupeng Fan^1^, Tangwei Huang^1^＆ Xiaolu Pan^1,4^


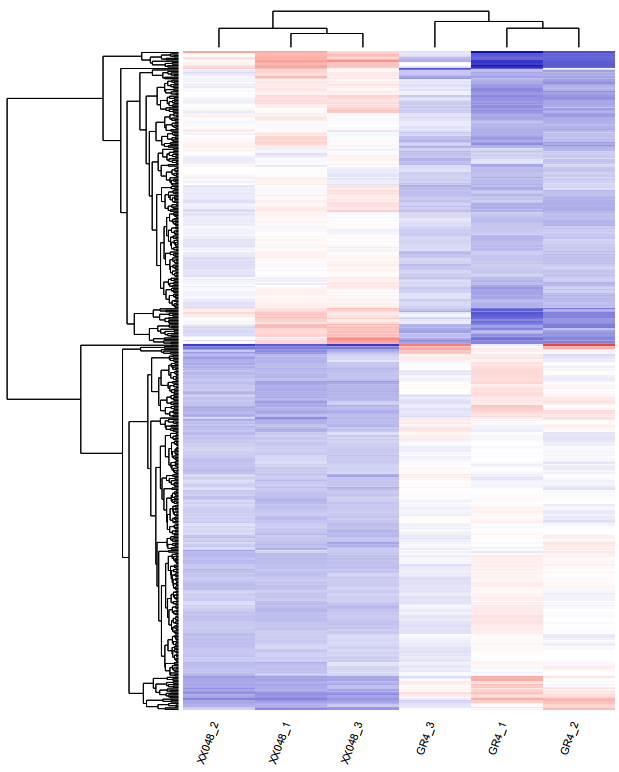

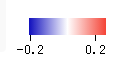


**GR4_2**

**GR4_1**

**GR4_3**

**XX048_3**

**XX048_1**

**XX048_2**


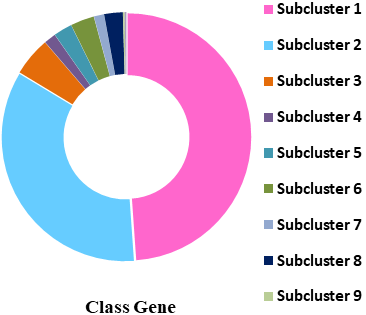


**Figure S1. Hierarchical clustering of the DEPs in XX048 and GR4.**


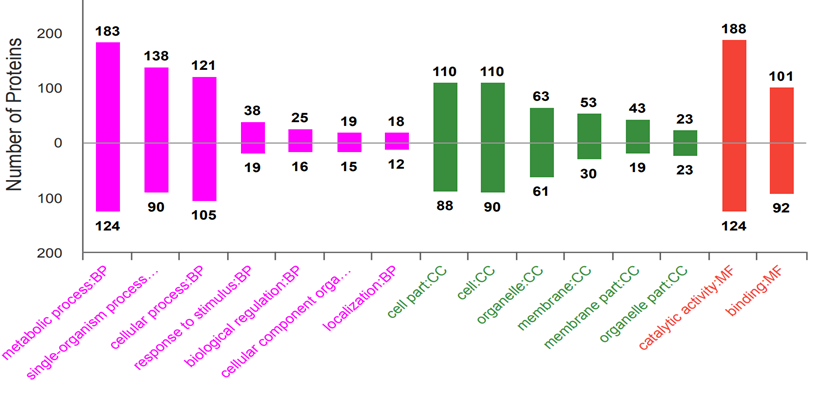


**Figure S2. GO analysis of the differentially expressed proteins in cassava leaves between XX048 and GR4.**

Expressed proteins involved in biological process, cellular component, and molecular function against the GO database. Above and below the ordinate zero are the number of up-regulated and down-regulated proteins, respectively.


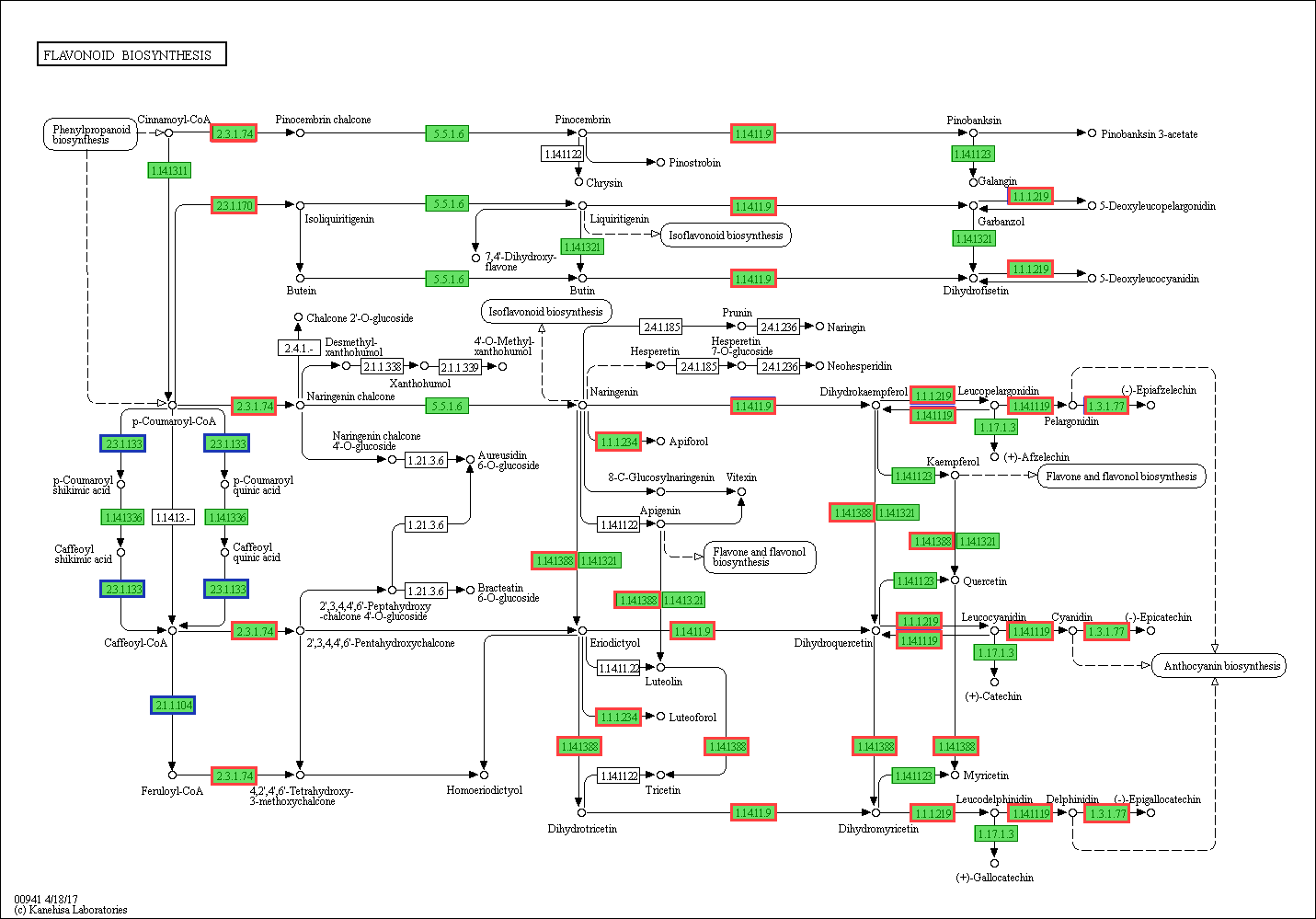


**Figure S3. Changes of the differentially expressed proteins in flavonoid biosynthesis pathway of XX048 and GR4 cassava leaves.** The proteins in red and blue are up-regulated and down-regulated expressed, respectively. The proteins in green background boxes are expressed at background level. The pathway map was generated using KEEG .
